# Effects of *Dictyophora indusiata* on Cognitive Impairment and Associated Neurobiological Changes in Chronic Mild Stress Mouse Model

**DOI:** 10.3390/ijms27188027

**Published:** 2026-09-09

**Authors:** Jatuporn Prathumtet, Tantima Kumlung, Warisada Sila-On, Sureewan Duangjit, Rawiwun Kaewamatawong, Wiwat Pichayakorn, Yaowared Sumanont, Orawan Monthakantirat, Utsana Puapermpoonsiri

**Affiliations:** 1 Department of Pharmaceutical Chemistry and Technology, Faculty of Pharmaceutical Sciences, Ubon Ratchathani University, Ubon Ratchathani 34190, Thailand; jatuporn.pr.63@ubu.ac.th (J.P.); sureewan.d@ubu.ac.th (S.D.); rawiwun.k@ubu.ac.th (R.K.); 2 Innovation in Drug and Extract of Agriculture (IDEA) Research Group, Ubon Ratchathani University, Ubon Ratchathani 34190, Thailand; tantima@tistr.or.th (T.K.); warisada.s@rsu.ac.th (W.S.-O.); 3 Thailand Institute of Scientific and Technological Research (TISTR), Pathum Thani 12120, Thailand; 4 Department of Manufacturing Pharmacy, College of Pharmacy, Rangsit University, Pathum Thani 12000, Thailand; 5 Department of Pharmaceutical Technology, Faculty of Pharmaceutical Sciences, Prince of Songkla University, Songkhla 90110, Thailand; wiwat.p@psu.ac.th; 6 Division of Pharmaceutical Chemistry, Faculty of Pharmaceutical Sciences, Khon Kaen University, Khon Kaen 40002, Thailand; yaosum@kku.ac.th (Y.S.); oramon@kku.ac.th (O.M.)

**Keywords:** *Dictyophora indusiata*, unpredictable chronic mild stress, oxidative stress, inflammation, cognitive performance

## Abstract

Chronic stress is associated with changes in cognitive performance involving oxidative stress and inflammatory responses. This study investigated the bioactive compounds, antioxidant and acetylcholinesterase (AChE) inhibitory activities, and potential effects of *Dictyophora indusiata* (*D. indusiata*) on cognitive performance in an unpredictable chronic mild stress (UCMS) mouse model. *D. indusiata* extracts contained phenolics, flavonoids, polysaccharides, and ergosterol. These extracts exhibited in vitro antioxidant and acetylcholinesterase (AChE) inhibitory activities. Oral administration of *D. indusiata* powder improved performance on cognitive tasks in UCMS mice, as assessed using the Y-maze test, novel object recognition test, and Morris water maze test. These improvements were accompanied by upregulated nuclear factor erythroid 2-related factor 2 (Nrf2) mRNA expression, downregulated Kelch-like ECH-associated protein 1 (Keap1) mRNA expression, increased superoxide dismutase (SOD) and catalase (CAT) activities, and reduced malondialdehyde (MDA) levels in the frontal cortex and hippocampus, suggesting potential involvement of the Keap1–Nrf2 signaling pathway. Furthermore, *D. indusiata* reduced mRNA expression of interleukin-1 beta (IL-1β), interleukin-6 (IL-6), and tumor necrosis factor-alpha (TNF-α). Taken together, these findings suggest potential involvement of antioxidant defense and inflammatory responses in the effects of *D. indusiata* on cognitive performance under chronic stress, while the relevance of in vitro AChE inhibition warrants further investigation.

## 1. Introduction

Chronic stress exposure has been demonstrated to play a crucial role in the progression of neurodegenerative diseases [1]. Growing evidence points to oxidative stress and inflammatory responses as key convergent mechanisms associated with stress-related brain dysfunction [2,3]. Chronic stress exposure can induce an imbalance between reactive oxygen species (ROS) and antioxidant defense, leading to excessive ROS production [2]. Oxidative stress may occur in tissues and induce brain damage, especially in the frontal cortex and hippocampus. These regions are part of a system responsible for numerous cognitive functions, emotional regulation, and memory consolidation [4]. Moreover, chronic stress can promote microglial activation and subsequently trigger the release of pro-inflammatory mediators, including interleukin-1β (IL-1β), interleukin-6 (IL-6), and tumor necrosis factor-α (TNF-α), in the brain. This response contributes to neuroinflammation, which may ultimately result in brain damage [3]. Numerous studies in rodent models have also shown that stress can disrupt levels of neurotransmitters such as monoamines and acetylcholine that are involved in arousal, emotion, and cognition [5,6]. Hence, modulation of oxidative stress and inflammatory responses represents a potential mechanism for mitigating the effects of chronic stress on cognitive function. Together, these processes have been associated with cognitive alterations under chronic stress conditions.

*Dictyophora indusiata* (*D. indusiata*) is an edible and medicinal fungus that can be found in the Asian regions. This mushroom is a member of the family Phallaceae in the class Agaricomycetes within the phylum Basidiomycetes. It is a rich source of protein, crude fiber, minerals, and vitamins [7]. *D. indusiata* also contains various bioactive compounds such as polysaccharides, terpenoids, alkaloids, and sterols that may be responsible for several medicinal properties [8]. Many studies have revealed that *D. indusiata* exhibits antioxidant, antihyperlipidemic, antitumor, and immunomodulatory effects [9,10,11,12]. *D. indusiata* has also been reported to have potential neurobiological effects, as it can increase the production of nerve growth factor (NGF) in astroglial cells [13]. It is also reported to protect cortical cells from excitotoxins and inhibit monoamine oxidase [14].

Ergosterol is one of the bioactive compounds isolated from *D. indusiata* [15] and has attracted considerable interest for its potential neurobiological effects. It has been reported that ergosterol from *Monascus anka* enhances the survival of cells damaged by H_2_O_2_, and reduces ROS levels within cells [16]. Moreover, ergosterol has demonstrated anti-inflammatory properties. Research has shown that ergosterol from *Antrodia camphorata* can reduce neuroinflammatory responses induced by lipopolysaccharide (LPS) in microglial cells and ICR mice [17]. Furthermore, ergosterol from *Auricularia polytricha* has been found to alleviate TNF-α-induced neuronal toxicity in HT-22 cells [18]. However, the potential effects of *D. indusiata* on cognitive performance under unpredictable chronic mild stress (UCMS) conditions in an animal model have not been elucidated.

Thus, this study aimed to evaluate the bioactive compounds, in vitro antioxidant activity, and in vitro acetylcholinesterase (AChE) inhibitory activity from *D. indusiata* extracts. The effects of *D. indusiata* powder on cognitive performance in male ICR mice exposed to UCMS were evaluated based on behavioral tests, antioxidant enzyme activity, and lipid peroxidation. In addition, the mRNA expression of genes related to antioxidant defense and inflammatory responses was further investigated. These results may enhance the existing understanding of the effects of *D. indusiata* on chronic stress-associated cognitive alterations and provide insights into its potential neurobiological effects.

## 2. Results

### 2.1. Total Phenolic, Flavonoid, and Polysaccharide Contents

Total phenolic, total flavonoid, and total polysaccharide contents of *D. indusiata* extracts in different solvents are summarized in Table 1. These contents were quantified as equivalents of gallic acid (GAE), quercetin (QE), and glucose, respectively. The results showed that the highest total phenolic and total flavonoid contents were found in the methanol extract (20.88 ± 0.04 mg GAE/g extract and 19.77 ± 0.36 mg QE/g extract). The highest total polysaccharide content was observed in the hydrophilic extracts, including the water, acid, and alkali extracts (382 to 995 mg glucose/g extract). These findings indicate that solvent selection plays a critical role in determining the phytochemical profile of *D. indusiata* extracts.

### 2.2. High-Performance Liquid Chromatography (HPLC) Analysis and Method Validation

**Figure 1:**
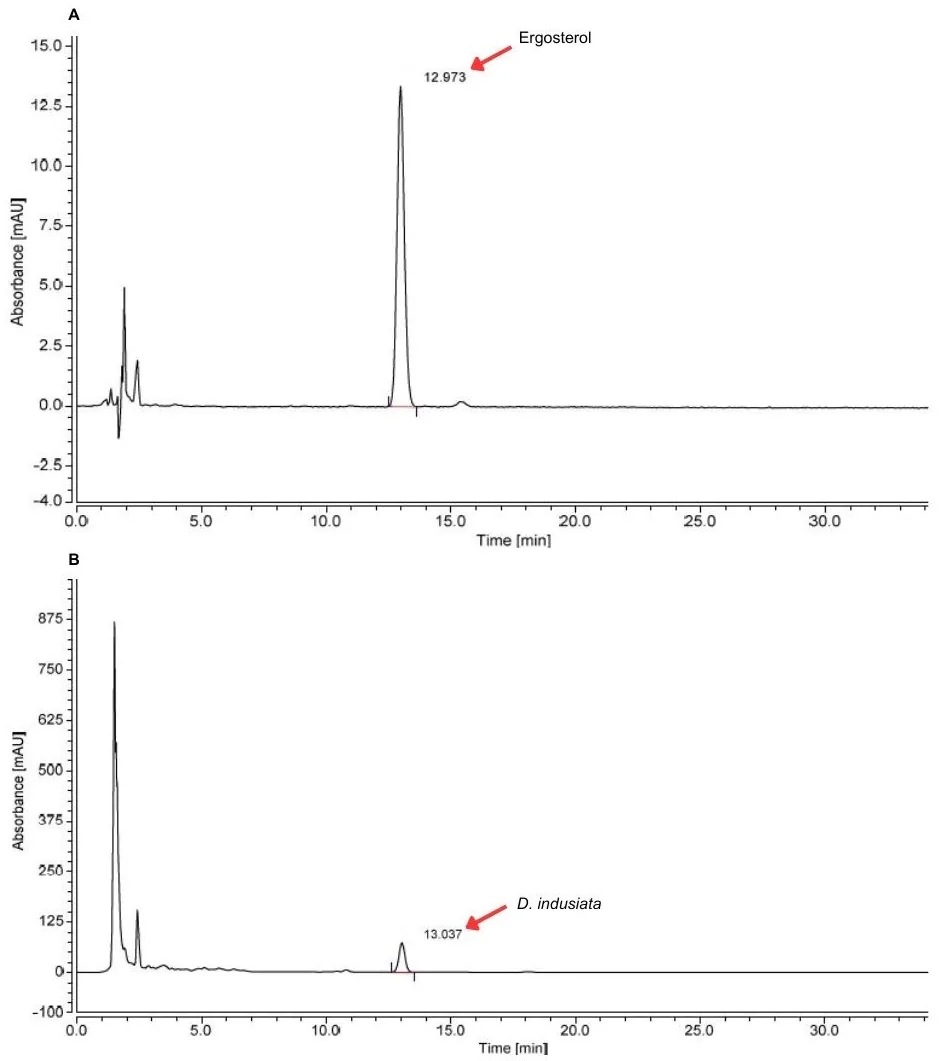
Chromatogram of ergosterol in *D. indusiata* extract: ergosterol (**A**) and *D. indusiata* extract (**B**).

Ergosterol, a characteristic fungal sterol with reported neuroprotective properties, was selected as the analytical marker for HPLC analysis of *D. indusiata* extracts. The analytical method demonstrated excellent validation performance, as shown in Table 2. Linearity of the method was established over the analytical range of 0.625–40 µg/mL. The equation was y = 0.4183x + 0.0071, and the correlation coefficient (R^2^) of ergosterol was 1, showing a good linear correlation. These complied with the AOAC Guidelines, which stated that the correlation coefficient should be >0.99 for acceptable linearity. The limit of detection (LOD) and limit of quantification (LOQ) values obtained from the calibration curve of ergosterol were 0.06 and 0.21 µg/mL, respectively. For precision, it was found that the within-day and between-day relative standard deviation (RSD) values were 1.46% and 4.09%, respectively. For accuracy, the percentage of recovery was found between 88.41% and 93.76%. It was within an acceptable range of 85–110% at a concentration of 0.01% that shows its suitability for quantification. Then, ergosterol in *D. indusiata* extract was also investigated. The HPLC chromatograms of ergosterol in *D. indusiata* extract are shown in Figure 1. The retention time was observed at 12.98 ± 0.01 min. The amount of ergosterol in *D. indusiata* extract was 0.37 ± 0.01 mg/g of extract.

### 2.3. Effect of D. indusiata on In Vitro Antioxidant Activity

The in vitro antioxidant activities of the *D. indusiata* extracts were determined by the 2,2′-azinobis(3-ethylbenzothiazoline-6-sulfonic acid) (ABTS) and 2,2-diphenyl-1-picrylhydrazyl (DPPH) radical scavenging assays, as shown in Table 3. Among all extracts, the methanol extract exhibited the strongest antioxidant activity in both the ABTS and DPPH radical scavenging assays (IC_50_ values of 2.28 ± 0.04 mg/mL in the ABTS radical scavenging assay and 4.54 ± 0.04 mg/mL in the DPPH radical scavenging assay), followed by the hydrophilic extracts and the hexane extract. These findings are consistent with the higher phenolic and flavonoid contents detected in the methanol extract, suggesting that phenolic constituents are major contributors to the antioxidant activity of *D. indusiata*.

### 2.4. Effect of D. indusiata on In Vitro Acetylcholinesterase (AChE) Inhibitory Activity

*D. indusiata* extracts were investigated for their AChE inhibitory activity, as shown in Table 4. The results revealed that the hydrophilic extracts exhibited no detectable AChE inhibitory activity. Among all extracts, the highest anti-AChE activity was observed in the hexane extract (IC_50_ value of 8.04 ± 0.22 mg/mL), followed by the methanol extract. These findings suggest that some relatively non-polar constituents of *D. indusiata* may be associated with the observed AChE inhibitory activity.

### 2.5. Effect of D. indusiata on Performance in Cognitive Tasks in Mice

**Figure 2:**
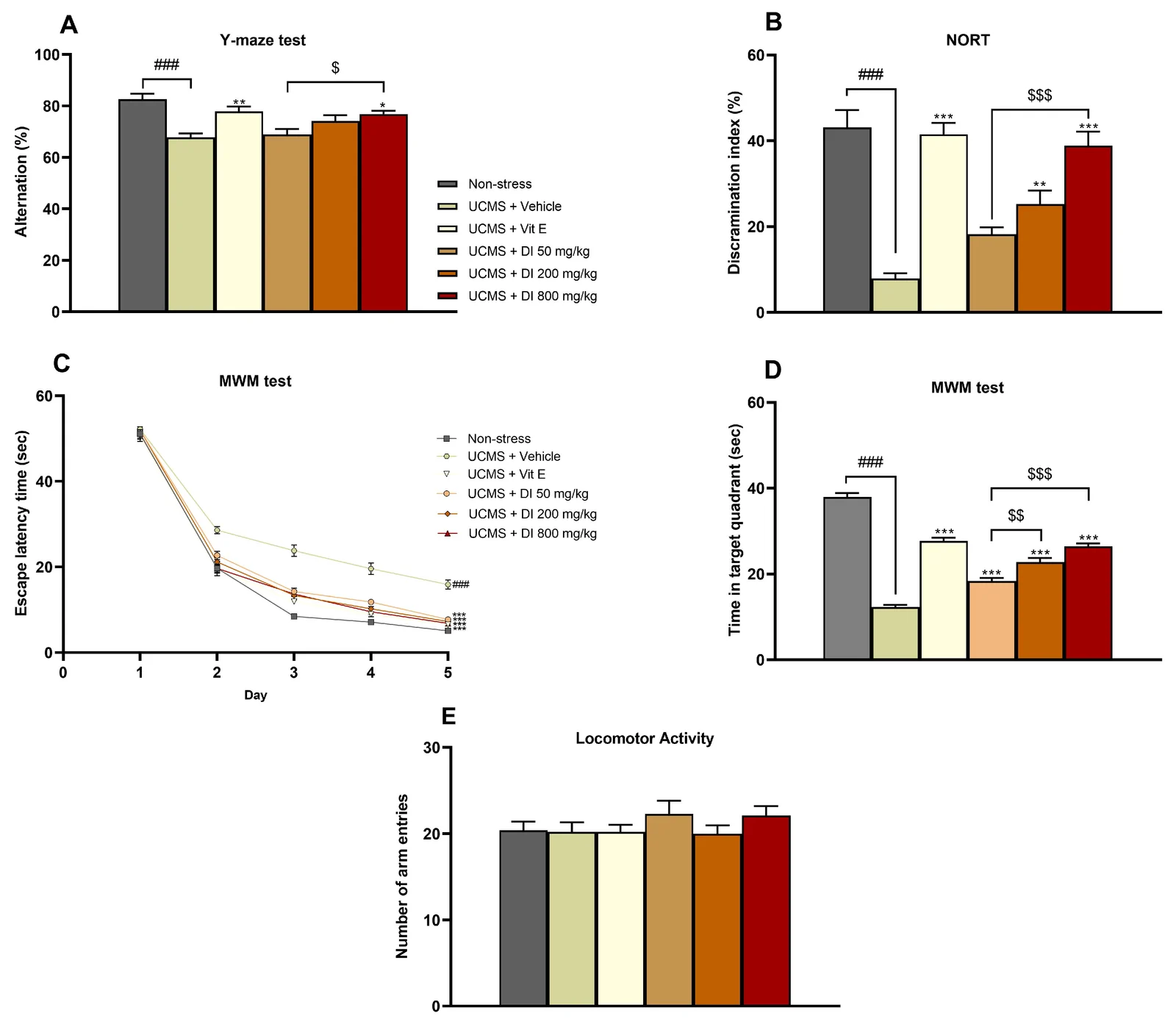
Effects of *D. indusiata* on cognitive performance in mice assessed using (**A**) the Y-maze test, (**B**) the NORT, (**C**) escape latency in the MWM test, and (**D**) time spent in the target quadrant in the MWM test. Locomotor activity was assessed by measuring the total number of arm entries in the Y-maze test (**E**). Data are shown as mean ± SEM (*n* = 10) (^###^ *p* < 0.001 compared to nonstressed mice; * *p* < 0.05, ** *p* < 0.01, *** *p* < 0.001 compared to UCMS model mice treated with vehicle; ^$^ *p* < 0.05, ^$$^ *p* < 0.01, ^$$$^ *p* < 0.001 compared to UCMS model mice treated with *D. indusiata* powder at a dose of 50 mg/kg), where DI is *D. indusiata* and Vit E is vitamin E.

After the in vitro studies, it was found that different solvent extractions produce different bioactive compounds that may have distinct neuroprotective mechanisms. Hence, *D. indusiata* powder was used for drug administration in a UCMS mouse model. The Y-maze test, novel object recognition test (NORT), and Morris water maze (MWM) test were conducted to evaluate cognitive performance in these mice. The results indicated that UCMS model mice treated with vehicle exhibited lower performance in the Y-maze test, the NORT, and the MWM test than nonstressed mice, indicating alterations in cognitive performance under UCMS exposure. For the results of the Y-maze test, UCMS model mice treated with *D. indusiata* powder at a dose of 800 mg/kg demonstrated a significantly higher percentage of alternation than that in UCMS model mice treated with vehicle, similar to the treatment with vitamin E (Figure 2A). Detailed statistical analyses are presented in Supplementary Table S2. For the NORT results, the percentage of discrimination index showed a significant improvement in UCMS model mice treated with *D. indusiata* powder at doses of 200 and 800 mg/kg compared to those in UCMS model mice treated with vehicle, similar to the treatment with vitamin E (Figure 2B). Statistical analyses are detailed in Supplementary Table S3. Furthermore, the escape latency time of mice to find the platform during the training day and the time spent in the target quadrant on the test day during the MWM test are shown in Figure 2C,D. Compared to UCMS model mice treated with vehicle, UCMS model mice treated with vitamin E and *D. indusiata* powder had shorter escape latency times from the second day to the last training day (day 5). On the testing day (day 6), the time spent in the target quadrant of UCMS model mice treated with *D. indusiata* powder at doses of 50, 200, and 800 mg/kg was significantly greater than that in UCMS model mice treated with vehicle, similar to the treatment with vitamin E. Detailed statistical analyses are reported in Supplementary Tables S4 and S5. These data suggest that each dose of *D. indusiata* powder was associated with improved performance in cognitive tasks in mice. Additionally, locomotor activity was assessed during the Y-maze test, as shown in Figure 2E. No significant differences were observed among the groups, suggesting that the observed differences in cognitive performance were unlikely to be due to changes in locomotor activity. Detailed statistical analysis is provided in Supplementary Table S1.

### 2.6. Effect of D. indusiata on Antioxidant Enzyme Activities and Lipid Peroxidation in the Mouse Brain

**Figure 3:**
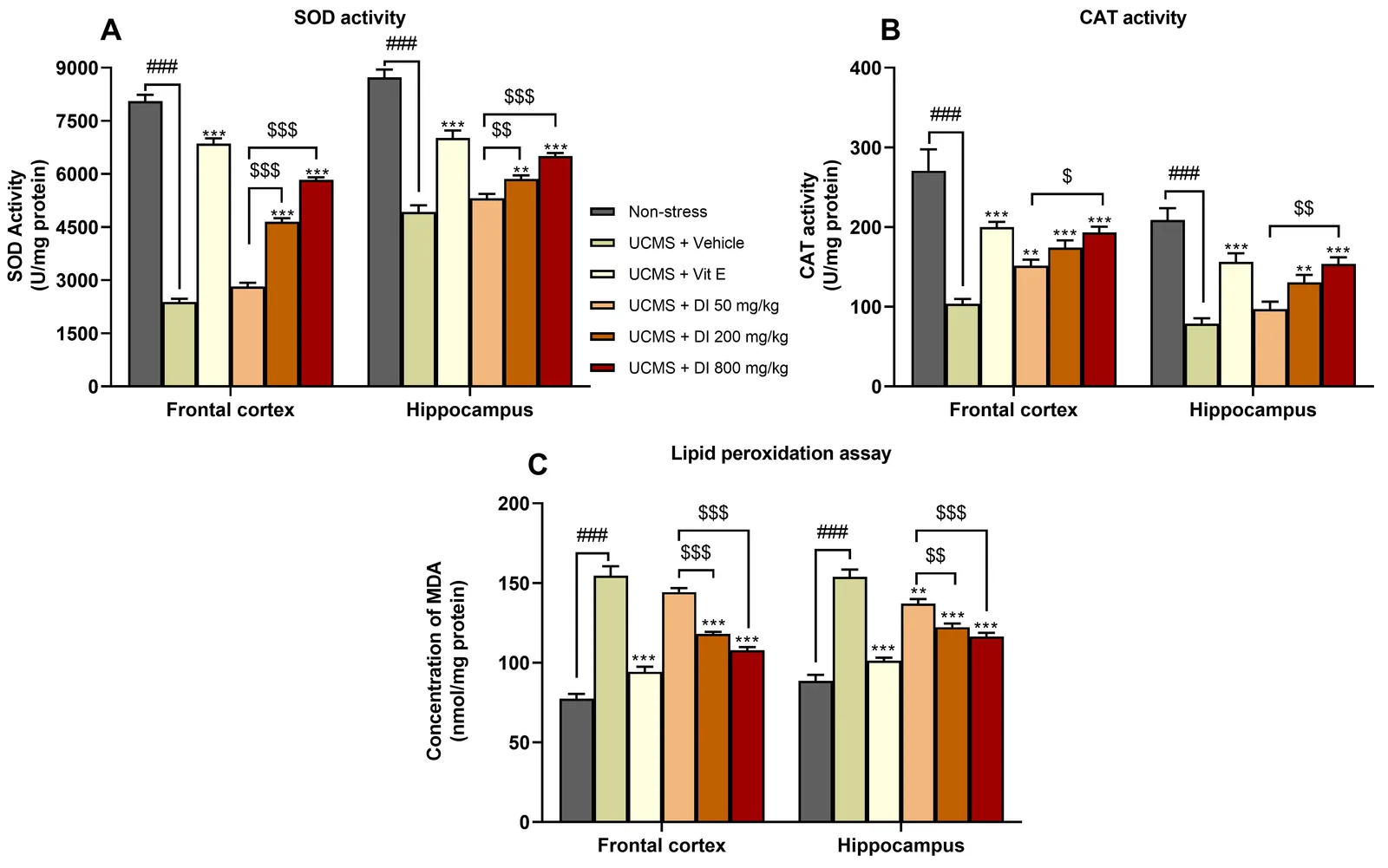
Effect of *D. indusiata* on antioxidant enzyme activities assessed by measuring (**A**) SOD and (**B**) CAT activities in the mouse brain. Lipid peroxidation was assessed by measuring (**C**) MDA levels. Data are shown as mean ± SEM (*n* = 5) (^###^ *p* < 0.001 compared to nonstressed mice; ** *p* < 0.01, *** *p* < 0.001 compared to UCMS model mice treated with vehicle; ^$^ *p* < 0.05, ^$$^ *p* < 0.01, ^$$$^ *p* < 0.001 compared to UCMS model mice treated with *D. indusiata* powder at dose of 50 mg/kg), where DI is *D. indusiata* and Vit E is vitamin E.

SOD and CAT activities were measured as indicators of antioxidant defense in the mouse brain, as illustrated in Figure 3A,B. SOD and CAT activities in the frontal cortex and hippocampus were significantly decreased in the UCMS model mice treated with vehicle when compared with the nonstressed mice. Administration of vitamin E significantly increased both SOD and CAT activities compared to those in UCMS model mice treated with vehicle. Additionally, SOD and CAT activities in the UCMS model mice treated with *D. indusiata* powder at doses of 200 and 800 mg/kg were significantly greater than those in the UCMS model mice treated with vehicle in both the frontal cortex and hippocampus. Moreover, the UCMS model mice treated with *D. indusiata* powder at a dose of 50 mg/kg showed a significant increase in CAT activity in the frontal cortex. See Supplementary Tables S6 and S7 for detailed statistical analyses.

Lipid peroxidation was also assessed by measuring malondialdehyde (MDA) levels in the frontal cortex and hippocampus (Figure 3C). The UCMS model mice treated with vehicle showed significantly higher MDA levels than nonstressed mice in both brain regions. In addition, treatment with vitamin E and *D. indusiata* powder at 200 and 800 mg/kg significantly reduced MDA levels in both the frontal cortex and hippocampus compared with the UCMS model mice treated with vehicle. Administration of *D. indusiata* powder at 50 mg/kg significantly reduced MDA levels in the hippocampus. See Supplementary Table S8 for detailed statistical analyses.

### 2.7. Effect of D. indusiata on the Expression of Genes Involved in Antioxidant Defense and Inflammatory Responses in the Mouse Brain

**Figure 4:**
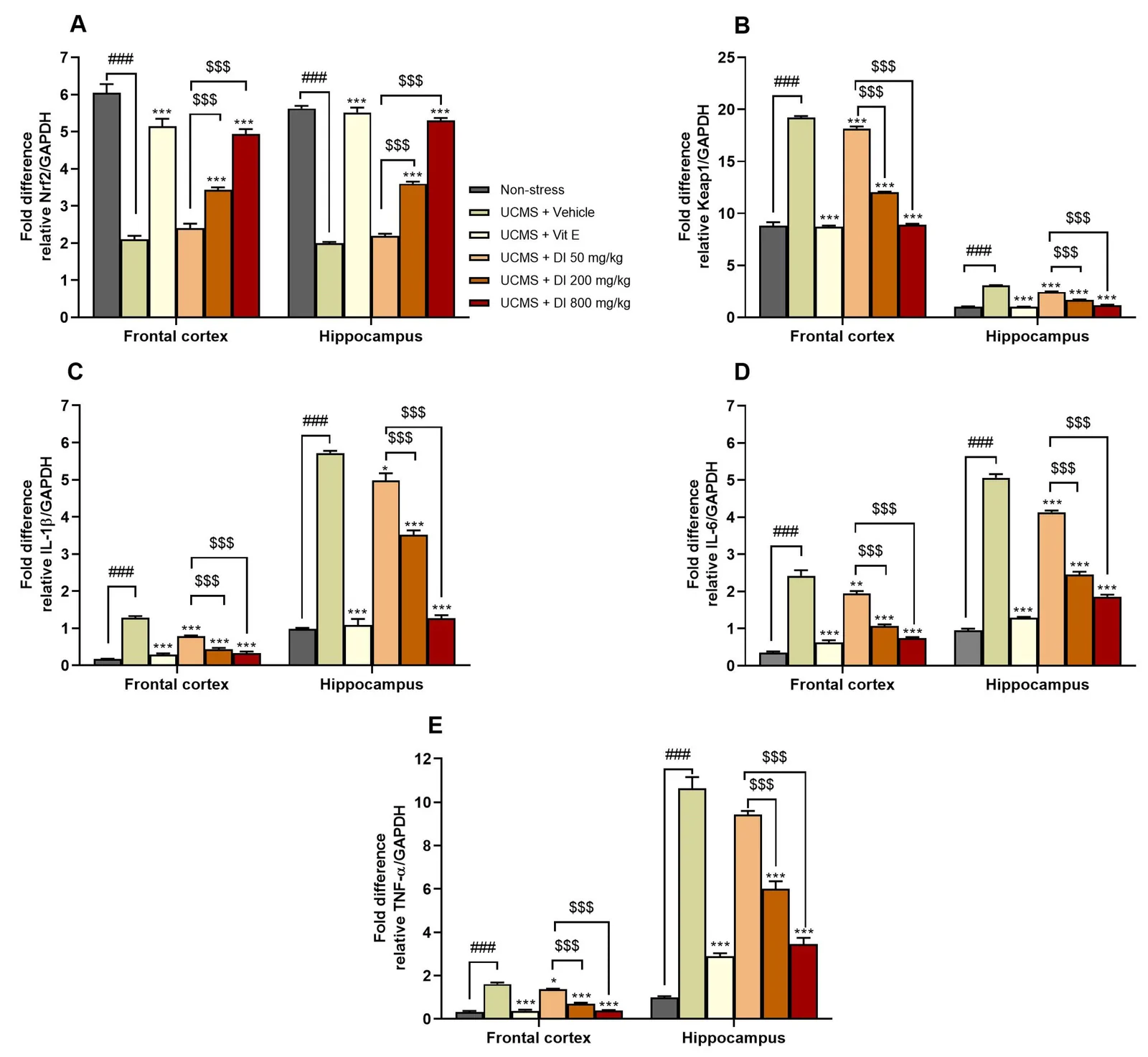
Effect of *D. indusiata* on the expression of genes involved in antioxidant defense and inflammatory responses in the mouse brain assessed by measuring (**A**) Nrf2, (**B**) Keap1, (**C**) IL-1β, (**D**) IL-6, and (**E**) TNF-α levels. Data are shown as mean ± SEM (*n* = 5) (^###^ *p* < 0.001 compared to nonstressed mice; * *p* < 0.05, ** *p* < 0.01, *** *p* < 0.001 compared to UCMS model mice treated with vehicle; ^$$$^ *p* < 0.001 compared to UCMS model mice treated with *D. indusiata* powder at dose of 50 mg/kg), where DI is *D. indusiata* and Vit E is vitamin E.

The mRNA expression of the antioxidant defense-related gene nuclear factor erythroid 2–related factor 2 (Nrf2) and Kelch-like ECH-associated protein 1 (Keap1) was measured in the mouse brains, as presented in Figure 4A,B. Mice subjected to UCMS showed reduced mRNA expression levels of Nrf2 and elevated mRNA expression levels of Keap1 compared to those in nonstressed mice. These changes were associated with UCMS exposure. Compared to UCMS model mice treated with vehicle, UCMS model mice treated with vitamin E and *D. indusiata* powder at doses of 200 and 800 mg/kg significantly increased Nrf2 mRNA expression in both the frontal cortex and hippocampus. In addition, UCMS model mice treated with vitamin E and *D. indusiata* powder at doses of 50, 200, and 800 mg/kg significantly decreased Keap1 mRNA expression in both the frontal cortex and hippocampus. Detailed statistical analyses are provided in Supplementary Tables S9 and S10.

Moreover, inflammatory response-related genes interleukin-1 beta (IL-1β), interleukin-6 (IL-6), and tumor necrosis factor-alpha (TNF-α) were also investigated in the mouse brain. As illustrated in Figure 4C–E, the UCMS model mice treated with vehicle exhibited greater mRNA expression levels of IL-1β, IL-6, and TNF-α than those in the nonstressed mice. Additionally, the intake of vitamin E and *D. indusiata* powder at doses of 200 and 800 mg/kg significantly reduced the mRNA expression levels of IL-1β, IL-6, and TNF-α compared to those in UCMS model mice treated with vehicle in both the frontal cortex and hippocampus. However, UCMS model mice treated with 50 mg/kg *D. indusiata* powder had lower IL-1β and IL-6 mRNA expression in both the frontal cortex and hippocampus, as well as TNF-α mRNA expression in the frontal cortex. Detailed statistical analyses are shown in Supplementary Tables S11–S13.

## 3. Discussion

Chronic stress is closely associated with oxidative stress that is believed to be one of the major factors contributing to the development of neurodegenerative diseases. Substantial evidence has demonstrated that prolonged exposure to stress may also induce neuroinflammation, leading to brain damage. Thus, searching for neuroprotective agents from natural sources is important for treating these diseases. *D. indusiata* is an edible mushroom that has high medicinal value. Hence, the main purpose of this study was to determine the bioactive compounds in *D. indusiata* extracts, as well as their in vitro antioxidant and AChE inhibitory activities. Additionally, the effects of *D. indusiata* powder on cognitive performance in male ICR mice exposed to UCMS and the associated neurobiological changes were investigated.

First, *D. indusiata* was extracted using water, acid, alkali, hexane, and methanol for phytochemical analysis via a colorimetric method. We found in this study that the hydrophilic extracts were rich in total polysaccharide content, whereas total flavonoids and total phenolic compounds were the major components in the methanol extract. This indicates that the type of solvent used can influence the extraction of bioactive compounds. In general, methanol is considered a universal solvent because it can dissolve polar, semi-polar, and non-polar compounds [19]. The extracts contain phenolic compounds and other substances with varying degrees of polarity. This makes methanol an effective solvent for both polar and less polar phenolic compounds. Moreover, many phenolic compounds are heat-sensitive and may degrade at high processing temperatures during extraction [20], resulting in a decrease in their levels after decoction. Meanwhile, water is the best solvent for polysaccharide extraction because most polysaccharides are polar and readily dissolve in hydrophilic solvents, such as water [20], while acids or bases have the ability to extract compounds by disrupting the cell wall and cleaving hydrogen bonds, leading to the release of intracellular polysaccharides [21]. Furthermore, ergosterol content in the methanol extract of *D. indusiata* was analyzed by HPLC. Our findings revealed that ergosterol (0.37 ± 0.01 mg/g extract) was detected in the methanol extract of *D. indusiata*. This finding supports the study by Zhang and coworkers, who isolated ergosterol derivatives from the ethyl acetate partition of *D. indusiata* ethanol extract and identified them using high-resolution electrospray ionization mass spectrometry (HRESIMS), 1D/2D nuclear magnetic resonance (NMR) spectroscopy, and ultraviolet (UV) spectroscopy [15]. As a whole, these bioactive compounds may exert neuroprotective effects through various mechanisms of action, which require further investigation.

In this study, the extract of *D. indusiata* was preliminarily screened for in vitro antioxidant activities. We demonstrated that the methanol extract had a stronger antioxidative effect than the other extracts in both the ABTS and DPPH radical scavenging assays. These results were supported by the previous experiment that the methanol extract had the highest total phenolic content. Venkatesan and coworkers reported that antioxidant activities were related to the phenolic compounds due to their redox properties, which allow them to act as singlet oxygen quenchers, reducing agents, and hydrogen donors [22]. The methanol extract of *D. indusiata* was also found to contain ergosterol, a compound reported to possess antioxidant properties that enhance yeast resistance to free radicals generated by tert-butyl hydroperoxide [23]. Furthermore, in vitro AChE inhibitory activity was evaluated. Our findings showed that the hexane and methanol extracts exhibited AChE inhibitory activity. Inhibition of this enzyme leads to an accumulation of acetylcholine (ACh) at the synaptic cleft. ACh is a neurotransmitter that plays a role in attention, cognition, learning, and memory [24]. However, given the relatively weak AChE inhibitory activity observed in vitro, AChE inhibition alone may not fully explain the cognitive improvements observed in vivo. Therefore, the effects of *D. indusiata* on cognitive performance may be associated with multiple biological processes, including antioxidant defense and inflammatory responses. These findings suggest that extracts obtained with different solvents may contain various bioactive compounds that show distinct effects on neurological functions.

The unpredictable chronic mild stress model is a robust experimental paradigm to investigate the effects of sustained stress. UCMS recapitulates salient elements of chronic stress-related disease and produces behavioral and molecular changes that reflect hallmarks of human stress-associated disorder [25]. Importantly, the most stress-sensitive brain regions include the frontal cortex and hippocampus that are extensively involved in cognition, emotional processing, and adaptive behavior [26]. Therefore, the UCMS mouse model was used to investigate cognitive performance under chronic stress in this study. Performance in cognitive tasks in mice was assessed using the Y-maze test, the NORT, and the MWM test. Typically, the percentage of alternation in the Y-maze test serves as an indicator of short-term spatial memory. Mice with intact short-term spatial memory were able to remember the previously visited arm and avoid entering it [27]. In the NORT, the percentage of the discrimination index was used to assess short-term nonspatial memory. Mice with good memory ability tended to spend more time exploring new objects because they could discriminate between familiar and new objects [28]. The MWM test was employed to assess spatial learning and memory abilities. The ability to learn can be seen in the training phase, while short-term memory ability could be observed one day after training or during the test phase [29]. Mice with intact spatial learning and memory abilities exhibited a decrease in escape latency and an increase in retention time in the quadrant where the platform had been placed. In the present study, we observed that vehicle treatment decreased the number of alternations, the discrimination index, and the swimming time in the target quadrant. These findings are consistent with previous studies showing that UCMS exposure is associated with changes in learning and memory performance in mice [30]. However, oral administration of *D. indusiata* powder significantly improved performance in behavioral tasks related to learning and memory, as evidenced by a greater percentage of alternation, discrimination index, and swimming time in the target quadrant. Consistent with a previous study, *D. indusiata* polysaccharide could alleviate arsenic-induced learning and memory deficits in rats. Rats treated with *D. indusiata* polysaccharides spent more time in the target quadrant than arsenic-treated rats in the MWM test [31]. These findings suggest that *D. indusiata* may have beneficial effects on performance in behavioral tasks related to learning and memory under chronic stress conditions. To ensure that results from the Y-maze test, the NORT, and the MWM test were not confounded by motor impairments, general locomotor activity was assessed, revealing no significant differences between groups. The consistent alterations observed across various behavioral tests, particularly those assessing spatial and recognition memory, indicate that cognitive functions were primarily affected. However, this interpretation should be made with caution because the present study did not include comprehensive assessments of anxiety-like behavior or motivation. These factors may influence performance in behavioral tasks, and, therefore, may have contributed to the observed behavioral effects. In addition, the absence of a normal control group receiving a high dose of *D. indusiata* limits our ability to specifically assess potential behavioral changes or adverse effects in normal animals. Further studies incorporating these additional behavioral assessments and an appropriate high-dose normal control group are warranted to clarify the effects of *D. indusiata* on cognitive performance and to further evaluate its safety profile.

Oxidative stress was evaluated by assessing both direct cellular damage and endogenous antioxidant capacity in the mouse frontal cortex and hippocampus. UCMS significantly increased lipid peroxidation, as demonstrated by elevated brain MDA levels, and simultaneously impaired antioxidant defenses, as indicated by decreased SOD and CAT enzymatic activities and reduced Nrf2/Keap1 signaling. These alterations indicate a disrupted redox balance and increased ROS generation, consistent with previous reports [30]. Mechanistically, SOD converts superoxide anions (O_2_^−^) into hydrogen peroxide (H_2_O_2_), which is subsequently decomposed into oxygen and water by CAT [32]. Treatment with *D. indusiata* powder significantly suppressed lipid peroxidation and restored SOD and CAT activities to levels comparable to those of vitamin E. This antioxidant protection may be driven by its bioactive constituents, specifically polyphenols, such as *p*-coumaric acid [33]. These compounds contain hydroxyl groups that directly scavenge ROS [34]. Furthermore, these findings align with observations by Zhang et al. [35], who reported that *D. indusiata* polysaccharides mitigate ROS and MDA accumulation while boosting SOD activity under oxidative stress.

Finally, the mRNA expression of antioxidant defense-related genes was examined to further explore the potential effects of *D. indusiata* in UCMS-exposed mice. Our findings showed lower Nrf2 mRNA expression and higher Keap1 mRNA expression in both the frontal cortex and hippocampus in UCMS-exposed mice. Following treatment with *D. indusiata* powder, Nrf2 mRNA expression increased and Keap1 mRNA expression decreased in these brain regions. These findings are consistent with those of Seo and coworkers [36], who reported that an antioxidant agent could disrupt the binding of Nrf2 to Keap1, resulting in an increase in the level of Nrf2 and a decrease in the level of Keap1. The Keap1–Nrf2 antioxidant response element (ARE) pathway is the major defense mechanism against free radicals and electrophilic chemicals. Nrf2 serves as the master transcription factor for regulating multiple antioxidant enzymes, and Keap1 acts as the master negative regulator of Nrf2 [37]. Under normal conditions, Nrf2 is located in the cytoplasm and is negatively regulated by Keap1. Keap1 binding prevents the nuclear translocation of Nrf2 and serves as the site of action for the cullin-dependent E3 ubiquitin ligase, which degrades Nrf2 through the ubiquitin–proteasome system [38]. When cells are exposed to ROS or antioxidant agents, the cysteine thiol group in Keap1 becomes oxidized, causing a conformational change that leads to the dissociation of Keap1 from Nrf2 [39]. Nrf2 then translocates to the nucleus and binds to small musculoaponeurotic fibrosarcoma (sMaf) proteins to form transcriptionally active heterodimers. The Nrf2-sMaf heterodimer also attaches to the ARE to initiate the transcription of target genes, including those of antioxidant and phase II detoxification enzymes, such as SOD, CAT, glutathione (GSH), heme oxygenase-1 (HO-1), and NAD(P)H:quinone oxidoreductase 1 (NQO1) [40]. Therefore, these findings may support the ex vivo results showing increased SOD and CAT activities in UCMS model mice treated with vitamin E and *D. indusiata* powder. Additionally, the mRNA expression of inflammatory response-related genes was also examined to further explore the potential effects of *D. indusiata* in UCMS-exposed mice. Our results showed that administration of *D. indusiata* powder reduced the increase in IL-1β, IL-6, and TNF-α mRNA expression observed in both the frontal cortex and hippocampus of UCMS model mice. This finding is consistent with another study that an aqueous extract of *D. indusiata* could suppress the secretion of inflammatory cytokines from macrophages [33]. Ruksiriwanich and coworkers also reported that the ethanolic extract of *D. indusiata* exhibited anti-inflammatory activity by decreasing the levels of nitric oxide (NO), IL-1β, IL-6, and TNF-α in the Raw 264.7 cell line [41]. In addition, evidence from the literature indicates that chronic stress can induce microglial activation [42]. Microglia are macrophages in the brain. They play a crucial role in the neuroinflammatory response [43]. Consequently, stress can activate microglia, resulting in the production of pro-inflammatory cytokines such as IL-1β, IL-6, and TNF-α in the frontal cortex and hippocampus [44]. However, the assessment of microglial and/or astrocytic activation, together with protein-level expression of inflammatory mediators, can provide stronger evidence for the presence and modulation of neuroinflammation. These assessments were not performed in the present study. Thus, the observed changes in IL-1β, IL-6, and TNF-α mRNA expression should be interpreted as changes in inflammatory responses rather than definitive evidence of neuroinflammation.

**Figure 5:**
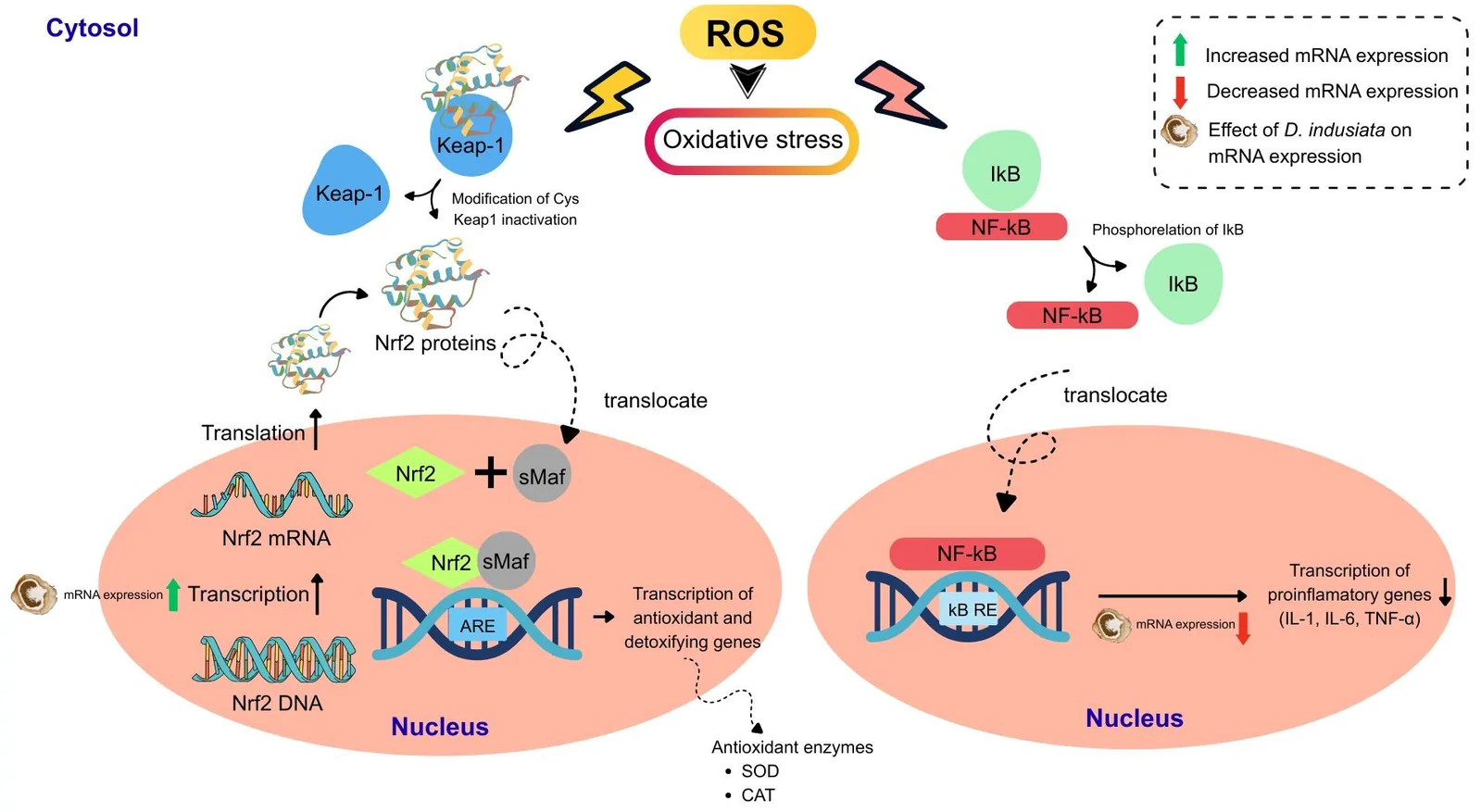
Proposed molecular mechanisms underlying the alleviation of cognitive impairment by *D. indusiata*.

Overall, *D. indusiata* may improve cognitive performance, which may be associated with changes in antioxidant defense and inflammatory responses. As illustrated in the proposed mechanism (Figure 5), the bioactive constituents of *D. indusiata* may exert complementary antioxidant effects by directly scavenging excessive reactive oxygen species (ROS) while simultaneously reinforcing endogenous antioxidant defense systems. The increase in Nrf2 mRNA expression together with the decrease in Keap1 mRNA expression suggests potential involvement of the Keap1–Nrf2/ARE signaling pathway, which may contribute to antioxidant defense. This interpretation is consistent with the increased SOD and CAT activities and reduced MDA levels observed following administration of *D. indusiata* powder. Furthermore, *D. indusiata* reduced the mRNA expression of the pro-inflammatory cytokines IL-1β, IL-6, and TNF-α, suggesting attenuation of inflammatory responses associated with prolonged stress exposure. NF-κB signaling was not directly evaluated in the present study; therefore, the observed reductions in these cytokines cannot be attributed specifically to inhibition of the NF-κB pathway. Nevertheless, previous studies have demonstrated that *D. indusiata*-derived preparations exert anti-inflammatory effects through modulation of NF-κB-related signaling, including inhibition of IκBα phosphorylation and NF-κB p65 nuclear translocation [45,46]. Accordingly, NF-κB represents a plausible, literature-supported upstream mechanism that may contribute to the anti-inflammatory effects observed in the present study, although direct involvement of this pathway remains to be experimentally confirmed. Together with the observed changes in antioxidant-related gene expression, these findings suggest a potential interplay between redox regulation and inflammatory responses that may contribute to the neuroprotective effects of *D. indusiata*. The present study provides evidence that the effects of *D. indusiata* on cognitive performance under chronic stress conditions may be associated with changes in antioxidant defense and inflammatory responses. These findings contribute to a better understanding of the potential biological effects of *D. indusiata* and provide a basis for further studies to clarify its effects on cognitive function.

## 4. Materials and Methods

### 4.1. Samples and Chemicals

The whole immature fruiting bodies of *D. indusiata* at the egg stage used in this study were collected as fresh mushroom samples and identified based on their morphological characteristics by Dr. Tantima Kumlung of the Thailand Institute of Scientific and Technological Research (TISTR), Thailand, prior to drying, extraction, and subsequent phytochemical and biological analyses. A voucher specimen (UBUM0002) was deposited in the Herbarium, Herbal Museum, Faculty of Pharmaceutical Sciences, Ubon Ratchathani University, Thailand.

### 4.2. Preparation of D. indusiata Extract

The *D. indusiata* powder was extracted with hot water, acid, alkali, methanol, and hexane at a ratio of 1:20 *w*/*v*. The hot water extract was prepared by boiling the powder with water at 95 °C for 2 h in a water bath, and the supernatant was collected. The supernatant was deproteinized with Sevag reagent and precipitated with cold ethanol. For acid and alkaline extraction, the powder was mixed with 1 M HCl and 1% NaOH at 55 °C for 7 h. The supernatant was neutralized and dialyzed in distilled water via a dialysis bag. The hot water, acid, and alkali extracts were then freeze-dried into powder. In addition, the organic solvent extraction was performed using hexane and methanol as solvents at 25 °C for 24 h. The samples were filtered and concentrated using a rotary vacuum evaporator. All the dried extracts were kept at −20 °C before use.

### 4.3. Determination of Total Phenolic, Flavonoid, and Polysaccharide Contents

The total phenolic content was determined using the Folin–Ciocalteu method as described by Singleton and Rossi [47], and gallic acid (Sigma-Aldrich, Beijing, China) was used as a standard. The samples were mixed with 10% (*v*/*v*) Folin–Ciocalteu reagent and 7.5% (*w*/*v*) sodium carbonate. The samples were allowed to incubate at room temperature for 30 min, and their absorbance was then measured at 765 nm using a microplate reader (Agilent BioTek, Winooski, VT, USA). The total flavonoid content was determined using the aluminum chloride method as reported by Laczkó-Zöld and coworkers [48], and quercetin (Sigma-Aldrich, Bengaluru, India) was used as a standard. The reaction mixture was prepared by mixing the samples with 2.5% (*w*/*v*) AlCl_3_, 10% (*w*/*v*) sodium acetate, and distilled water. It was incubated for 15 min before absorbance was taken at 430 nm using a microplate reader. The total polysaccharide content was determined using the phenol-H_2_SO_4_ method according to a modified procedure of Ibrahim [49], and D-glucose (CARLO ERBA Reagents GmbH, Cornaredo, Italy) was used as a standard. A 5% phenol solution and concentrated H_2_SO_4_ were added to the samples. After mixing, their absorbance was measured at 490 nm using a microplate reader.

### 4.4. HPLC Analysis and Method Validation

Ergosterol (Sigma-Aldrich, St. Louis, MO, USA) was used as the standard in this study. *D. indusiata* extract was analyzed by HPLC equipped with a quaternary pump, column oven, autosampler, and UV/VIS detector (Thermo Scientific Dionex, Germering, Germany). Chromatography was performed under isocratic conditions with a total run time of 45 min. The HPLC column used was a Reprosil-Pur Basic C18, 5 µm, 150 × 4.6 mm (Dr-Malsch, Ammerbuch, Germany). The mobile phase consisted of acetonitrile and methanol (70:30 *v*/*v*). The flow rate of the system was 1.0 mL/min. The column temperature was maintained at 35 °C in a column oven. The injection volume was 20 µL, and UV detection was used at 280 nm. Then, *D. indusiata* extract was prepared by dissolving *D. indusiata* powder (0.5 g) in methanol (10 mL). It was also sonicated at 37 °C for 90 min. Before injection into the HPLC system, the samples were filtered using a 0.45 µm nylon syringe filter. The content of ergosterol in *D. indusiata* extract was determined using a standard calibration curve. Moreover, the analytical method was validated following ICH Q2 (R2) guidelines. Precision was evaluated based on %RSD for both intra-day and inter-day determinations. Accuracy was measured by the percentage recovery at three standard concentrations. Linearity was evaluated by linear regression analysis of the calibration curve to determine the coefficient of determination (R^2^). LOD and LOQ were also validated.

### 4.5. In Vitro Antioxidant Activity

#### 4.5.1. ABTS Radical Scavenging Assay

The ABTS radical scavenging assay was carried out according to the modified method of Mamah and coworkers [50]. The ABTS^•+^ solution was prepared using 7 mM ABTS (Sigma-Aldrich, St. Louis, MO, USA) and 2.45 mM potassium persulfate with a ratio of 1:0.5 in the dark at room temperature for 12–16 h. It was diluted with ethanol to get an absorbance value of 0.70 ± 0.02. Briefly, 180 µL of the ABTS^•+^ mixture was added to 20 µL of the sample in a 96-well plate. This mixture was incubated for 6 min. The absorbance was measured at 734 nm using a microplate reader. Trolox (Sigma-Aldrich, Buchs, Switzerland) was used as a positive control. Then, the results were expressed as IC_50_ values that were obtained from a calibration curve of concentration and percentage inhibition.

#### 4.5.2. DPPH Radical Scavenging Assay

The DPPH radical scavenging assay was carried out according to the modified method of Tailor and Mamah [50,51]. Briefly, the mixture was produced by mixing 20 µL of the sample and 180 µL of DPPH solution (Merck, Darmstadt, Germany). After 30 min of incubation in the dark, the absorbance was measured at 517 nm using a microplate reader. Trolox was used as a positive control. Then, the results were expressed as IC_50_ values obtained from a concentration-percentage radical inhibition calibration curve.

### 4.6. In Vitro AChE Inhibitory Activity

The AChE inhibitory activity was carried out according to the modified method of Ellman and Ingkaninan [52,53]. Galanthamine hydrobromide (Sigma-Aldrich, St. Louis, MO, USA) was used as a positive control in this study. Acetylthiocholine iodide (ATCI, Sigma-Aldrich, Beijing, China) was used as the substrate, and AChE from electric eel (Sigma-Aldrich, St. Louis, MO, USA) was used as the enzyme source in the reaction. Briefly, the reaction mixture was composed of 50 mM Tris-HCl buffer, the sample, and 0.28 Units/mL AChE solution, and 3 mM 5,5′-dithio-bis-(2-nitrobenzoic acid) (DTNB, Sigma-Aldrich, Beijing, China) was placed in a 96-well plate. After 15 min of incubation, 1 mM ATCI was added to initiate the reaction, and its absorbance at 405 nm was read every 30 s for 5 min. Then, the results were expressed as IC_50_ values that were obtained from a calibration curve of concentration and percentage of AChE inhibition.

### 4.7. Animals

Male ICR mice (5 weeks old, 20–30 g) were purchased from the Northeast Laboratory Animal Center, Khon Kaen, Thailand. The experiments were conducted in accordance with the Guiding Principles for the Care and Use of Animals (NIH Publications #80-23, revised in 2011) and the ARRIVE 2.0 Guideline. All procedures were approved by the Animal Ethics Committee of Khon Kaen University, Khon Kaen, Thailand (approval No. IACUC-KKU-37/65). The animals were housed in stainless steel cages containing wood chip bedding with free access to food and water. The room conditions of the animals were maintained at a temperature of 22 ± 2 °C with a relative humidity of 45% ± 2% and a light–dark cycle of 12 h. This housing was located in the Laboratory Animal Unit of the Faculty of Pharmaceutical Sciences at Khon Kaen University, Thailand.

### 4.8. Unpredictable Chronic Mild Stress (UCMS) Procedure

After adaptation and grouping, mice were subjected to UCMS procedures. These procedures included the following: one period of food and water deprivation (18 h), two periods of the cage tilted at 45 degrees (12 h), two periods of restricted access to food with five micropellets (1 h each), two periods of exposure to an empty water bottle (3 h each), one period of a wet cage (21 h), two periods of prolonged light exposure (36 h), two periods of intermittent sound exposure (3 h and 5 h), and two periods of paired caging (2 h each) [54]. All of these stressors were randomly scheduled for one week during the day–night period, and the procedure was repeated for 6 weeks.

### 4.9. Experimental Design and Drug Administration

Mice were randomly divided into six groups (*n* = 10 per group). Group 1 (normal control group) served as nonstressed mice treated with vehicle (0.5% sodium carboxymethyl cellulose; SCMC, HiMedia laboratories, Mumbai, India). Group 2 (negative control group) served as UCMS model mice treated with vehicle (0.5% SCMC). Group 3 (positive control group) served as UCMS model mice treated with vitamin E (Sigma-Aldrich, St. Louis, MO, USA) in 0.5% SCMC (100 mg/kg). Groups 4–6 (treatment group) served as UCMS model mice treated with *D. indusiata* powder at doses of 50, 200, and 800 mg/kg, respectively. The treatments were given orally once daily at 8:00 am for 3 weeks after day 21 and were administered 1 h before behavioral testing.

### 4.10. Behavioral Studies

#### 4.10.1. Y-Maze Test

The Y-maze test was used to evaluate short-term spatial memory. The equipment in this study included a Y-shaped structure with three arms of equal size (40 cm long × 3 cm wide × 12 cm high), with a 120° angle between each of the 2 arms. Each mouse was positioned at the end of one of the arms and allowed to move freely within the maze for 5 min. The number and sequence of arm entries were recorded. The maze was cleaned with 70% ethanol and dried using paper towels before the next mouse was tested [26]. Alternations were then defined as successive entries into three different arms (e.g., ABC, ACB, CBA, CAB, BCA, and BAC, but not CBC). The percentage of alternation was calculated according to Equation (1).

(1) % Alternation = [(Number of alternations)/(Total arm entries − 2)] × 100

#### 4.10.2. Novel Object Recognition Test (NORT)

The NORT was conducted to assess short-term nonspatial memory, which is based on the tendency of mice to discriminate between familiar and novel objects. The test comprised three phases: the habituation phase, the sample phase, and the test phase. In the habituation phase, each mouse was placed in an empty plastic black box (50 cm long × 50 cm wide × 40 cm high) for 15 min to adapt to the new environment. After a 24 h period, the sample phase testing began. During this phase, each mouse was allowed to move freely within the black plastic box, which contained two identical objects (familiar objects), for 5 min. The test phase was conducted 30 min after the sample phase. In this phase, one of the objects was replaced with a novel object. Each mouse was placed in the box once again and given 5 min to explore the objects. The time spent exploring the objects, recorded when the mouse’s head was facing, touching, or sniffing the object, was noted. Both the plastic black box and the objects were cleaned with 70% ethanol and dried with paper towels [26]. Subsequently, the percentage discrimination index was calculated according to Equation (2).

(2) % Discrimination index = [(TN − TF)/(TN + TF)] × 100

where TN and TF are the times spent exploring the novel and familiar objects, respectively.

#### 4.10.3. Morris Water Maze (MWM) Test

The MWM test was used to assess learning and memory function. The apparatus was composed of a circular black pool with a diameter of 75.8 cm and a height of 25 cm. The circular black pool was divided into four quadrants, with the platform (12 cm long × 4 cm wide × 14 cm high) located in the center of quadrant 1 and submerged 1.5 cm below the water surface. The MWM test consisted of a training phase and a test phase. During the training phase, each mouse was placed in the pool at four different starting points (in different quadrants) and allowed to swim for 60 s. The mice were trained to find the hidden platform, and the time required to reach it was recorded as “escape latency”. If a mouse failed to find the platform within 60 s, the researcher guided the mouse to the platform. After five days of training, the test phase was conducted. The platform was removed, and each mouse was placed in the water at any starting point and allowed to swim for 60 s. The time spent swimming in the target quadrant (retention time) was recorded [55]. A shorter escape latency and a longer retention time indicated greater cognitive abilities.

#### 4.10.4. Locomotor Activity

Locomotor activity was evaluated using the Y-maze test to determine whether the treatments affected the general movement of the mice. Each mouse was placed in one arm and allowed to explore freely for 5 min. The total number of arm entries was recorded, and the maze was cleaned with 70% (*w*/*v*) ethanol after each test.

### 4.11. Measurement of Superoxide Dismutase (SOD) and Catalase (CAT) Activities

SOD and CAT are the most important antioxidative enzymes. SOD catalyzes the dismutation of O_2_^−^ into H_2_O_2_ and O_2_, while CAT catalyzes the decomposition of H_2_O_2_ into O_2_ and H_2_O. Their activities were investigated using commercially available kits from Sigma-Aldrich (Sigma-Aldrich, St. Louis, MO, USA) following the manufacturer’s instructions. In this study, samples were prepared by homogenizing brain tissue (from the frontal cortex or hippocampus) with 5 mM PBS (pH 7.4) and collecting the supernatants. The activities of SOD and CAT were determined using a colorimetric method, and their absorbance was measured. Additionally, SOD was used at concentrations ranging from 0.001 to 50 U/mL, and CAT was used at concentrations ranging from 0.0125 to 0.075 mM as positive controls for SOD and CAT activities, respectively. The resulting SOD and CAT activities were normalized to their respective total protein concentrations, which were determined using the Bradford protein assay as previously reported [26].

### 4.12. Measurement of MDA Levels

Malondialdehyde (MDA) levels in brain homogenates were assessed as an indicator of lipid peroxidation using the thiobarbituric acid reactive substances (TBARS) assay, adapted from a previously described method [26]. Briefly, the frontal cortex or hippocampus was homogenized in 5 mM phosphate-buffered saline (PBS, pH 7.4) at a 1:10 (*w*/*v*) ratio. The homogenate was mixed with 10% trichloroacetic acid (TCA; Sigma-Aldrich, St. Louis, MO, USA) and centrifuged at 8000× *g* for 15 min at 4 °C. The supernatant was then mixed with 0.8% 2-thiobarbituric acid (TBA; Sigma-Aldrich, St. Louis, MO, USA) and heated at 100 °C for 15 min. After cooling, 100 µL of the reaction mixture was transferred to a 96-well plate, and absorbance was measured at 532 nm. MDA (Sigma-Aldrich, St. Louis, MO, USA) at a concentration range from 0.001 to 0.075 µM was used as a positive control. The results were expressed as nmol of MDA per mg of protein. Protein concentration was determined using the Bradford method.

### 4.13. Quantitative Real-Time Polymerase Chain Reaction (qPCR)

After dissecting the frontal cortex and hippocampus, qPCR was performed to examine the expression levels of oxidative stress- and inflammatory-related genes in the brain, including Nrf2, Keap1, IL-1β, IL-6, and TNF-α, in accordance with the manufacturer’s instructions. Additionally, glyceraldehyde 3-phosphate dehydrogenase (GAPDH) was used as a housekeeping gene. The primer sequences used for qRT-PCR analysis are listed in Table 5. First, total RNA was extracted from brain tissue by homogenization with TRIzol^®^ reagent (Invitrogen^TM^, Thermo Fisher Scientific, Waltham, MA, USA) and subsequently converted to cDNA using a Superscript III First-Strand Synthesis Kit (Invitrogen^TM^, Thermo Fisher Scientific, Waltham, MA, USA). Next, qPCR was conducted using SsoAdvanced™ Universal SYBR^®^ Green Supermix (Bio-Rad, Hercules, CA, USA) with a preheating cycle at 95 °C for 3 min, followed by denaturation, annealing (61.40 °C for Nrf2, 59 °C for Keap1, 55 °C for IL-1β, 64.50 °C for IL-6, 59 °C for TNF-α, and 56 °C for GAPDH), and extension reaction cycles. All steps were performed in a Bio-Rad CFX96 Touch Real-Time PCR Detection System (Bio-Rad, Hercules, CA, USA). mRNA expression was analyzed using Bio-Rad CFX Manager 3.1 software to determine the threshold cycle (C_T_). The results are expressed as the fold difference, calculated using the 2^−ΔΔCT^ method.

**Table 5:** Primer sequences used for qRT-PCR analysis.

Genes		Primer Sequences	Product Length
*Nrf2*	Forward	5′-CAGTGCTCCTATGCGTGAA-3′	109 bp
	Reverse	5′-GCGGCTTGAATGTTTGTC-3′	
*Keap1*	Forward	5′-CATCCACCCTAAGGTCATGGA-3′	291 bp
	Reverse	5′-GACAGGTTGAAGAACTCCTCC-3′	
*IL-1β*	Forward	5′-GACAGCAAGTGATAGGCC-3′	95 bp
	Reverse	5′-CGTCGGCAATGTATGTGTTGG-3′	
*IL-6*	Forward	5′-CTTCCATCCAGTTGCCTTCTT-3′	142 bp
	Reverse	5′-AATTAAGCCTCCGACTTGTGAAG-3′	
*TNF-α*	Forward	5′-GCCTCTTCTCATTCCTGCTTG-3′	115 bp
	Reverse	5′-CTGATGAGAGGGAGGCCATT-3′	
*GAPDH*	Forward	5′-TGGAGAAACCTGCCAAGTATGA-3′	452 bp
	Reverse	5′-TGGAAGAATGGGAGTTGCTGT-3′	

### 4.14. Statistical Analysis

In vitro experiments, data were expressed as mean ± standard deviation (SD). The results from in vivo experiments were expressed as the mean ± standard error of mean (SEM). Statistical significance was determined using Student’s *t*-test or one-way analysis of variance (ANOVA), followed by Tukey’s post hoc test. In addition, escape latency during the 5-day training period of the MWM test was analyzed using two-way repeated-measures ANOVA, with treatment group and training day as factors. For all statistical analyses, significance levels were set at *p*-value < 0.05, 0.01, and 0.001. The analyses were conducted using IBM^®^ SPSS^®^ Statistics 29 (IBM Corp.©, Armonk, NY, USA).

## 5. Conclusions

*D. indusiata* is an attractive source of bioactive compounds, including total phenolics, flavonoids, polysaccharides, and ergosterol, which are abundant in different extractions. The extracts demonstrated in vitro antioxidant activity, along with in vitro AChE inhibitory activity, indicating their potential biological activities. In the animal study, the administration of *D. indusiata* powder improved performance on cognitive tasks in UCMS-exposed mice. These effects may be related to changes in antioxidant defense in the brain, as indicated by altered Nrf2 and Keap1 mRNA expression, increased SOD and CAT activities, and reduced MDA levels, together with changes in inflammation-related gene expression. Collectively, these findings suggest that *D. indusiata* may have potential to support cognitive performance under chronic stress conditions and warrant further investigation to further clarify its effects. These findings may also contribute to broader efforts toward the Sustainable Development Goals (SDGs) by expanding scientific knowledge on natural bioactive materials relevant to cognitive health and well-being.

## Figures and Tables

**Table 1:** Bioactive compounds content of *D. indusiata* extracts.

Samples	Total Phenolic (mg GAE/g Extract)	Total Flavonoid (mg QE/g Extract)	Total Polysaccharide (mg Glucose/g Extract)
Water extract	3.70 ± 0.06	2.44 ± 0.41	538.28 ± 2.41
Acid extract	15.10 ± 0.14	3.72 ± 0.10	995.78 ± 3.47
Alkali extract	14.67 ± 0.13	2.52 ± 0.12	382.44 ± 2.54
Hexane extract	0.88 ± 0.04	0.48 ± 0.09	24.67 ± 2.10
Methanol extract	20.88 ± 0.04	19.77 ± 0.36	145.78 ± 2.55

**Table 2:** Validation results for the analysis of ergosterol by HPLC.

Parameters	Ergosterol
Range (µg/mL)	0.625–40
Linearity	
(1) R^2^	1
(2) Equation	y = 0.4183x + 0.0071
Accuracy (% recovery)	88.41–93.76
Precision (% RSD)	
(1) Within-day	1.46
(2) Between-day	4.09
LOD (µg/mL)	0.06
LOQ (µg/mL)	0.21
Retention time (minutes)	12.98 ± 0.01
Wavelength detection (nm)	280

**Table 3:** In vitro antioxidant activity of *D. indusiata* extracts.

Samples	Antioxidant Activity (IC_50_; mg/mL)	
	ABTS Radical Scavenging Assay	DPPH Radical Scavenging Assay
Water extract	5.89 ± 0.16	6.57 ± 0.20
Acid extract	7.01 ± 0.11	22.80 ± 0.22
Alkali extract	2.70 ± 0.04	7.52 ± 0.26
Hexane extract	31.18 ± 0.06	43.53 ± 0.40
Methanol extract	2.28 ± 0.04	4.54 ± 0.04
Trolox	214.08 ± 5.80 µM	296.29 ± 0.33 µM

**Table 4:** In vitro AChE inhibitory activity of *D. indusiata* extracts.

Samples	AChE Inhibitory Activity (IC_50_; mg/mL)
Water extract	ND
Acid extract	ND
Alkali extract	ND
Hexane extract	8.04 ± 0.22
Methanol extract	22.47 ± 0.60
Galantamine	3.64 ± 0.12 µM

ND, not detected.

## Data Availability

The original contributions presented in this study are included in the article/Supplementary Materials. Further inquiries can be directed to the corresponding author.

## Supplementary Materials

The following supporting information can be downloaded at https://www.mdpi.com/article/10.3390/ijms27188027/s1.

## Abbreviations

The following abbreviations are used in this manuscript:

DI	*Dictyophora indusiata*
UCMS	Unpredictable chronic mild stress
ROS	Reactive oxygen species
RSD	Relative standard deviation
LOD	Limit of detection
LOQ	Limit of quantification
ABTS	2,2′-azinobis(3-ethylbenzothiazoline-6-sulfonic acid)
DPPH	2,2-diphenyl-1-picrylhydrazyl
AChE	Acetylcholinesterase
NORT	Novel object recognition test
MWM	Morris water maze
SOD	Superoxide dismutase
CAT	Catalase
Nrf2	Nuclear factor erythroid 2–related factor 2
Keap1	Kelch-like ECH-associated protein 1
GAPDH	Glyceraldehyde 3-phosphate dehydrogenase
IL-1β	Interleukin-1 beta
IL-6	Interleukin-6
TNF-α	Tumor necrosis factor-alpha

## References

[1] Graybeal C., Kiselycznyk C., Holmes A. (2012). Stress-Induced Deficits in Cognition and Emotionality: A Role for Glutamate. Curr. Top. Behav. Neurosci..

[2] Baierle M., Nascimento S.N., Moro A.M., Brucker N., Freitas F., Gauer B., Durgante J., Bordignon S., Zibetti M., Trentini C.M. (2015). Relationship between Inflammation and Oxidative Stress and Cognitive Decline in the Institutionalized Elderly. Oxidative Med. Cell. Longev..

[3] Decandia D., Gelfo F., Landolfo E., Balsamo F., Petrosini L., Cutuli D. (2023). Dietary Protection against Cognitive Impairment, Neuroinflammation and Oxidative Stress in Alzheimer’s Disease Animal Models of Lipopolysaccharide-Induced Inflammation. Int. J. Mol. Sci..

[4] Li M., Long C., Yang L. (2015). Hippocampal-Prefrontal Circuit and Disrupted Functional Connectivity in Psychiatric and Neurodegenerative Disorders. Biomed. Res. Int..

[5] Ma S., Hangya B., Leonard C.S., Wisden W., Gundlach A.L. (2018). Dual-Transmitter Systems Regulating Arousal, Attention, Learning and Memory. Neurosci. Biobehav. Rev..

[6] Mineur Y.S., Mose T.N., Vanopdenbosch L., Etherington I.M., Ogbejesi C., Islam A., Pineda C.M., Crouse R.B., Zhou W., Thompson D.C. (2022). Hippocampal Acetylcholine Modulates Stress-Related Behaviors Independent of Specific Cholinergic Inputs. Mol. Psychiatry.

[7] Habtemariam S. (2019). The Chemistry, Pharmacology and Therapeutic Potential of the Edible Mushroom *Dictyophora indusiata* (Vent Ex. Pers.) Fischer (Synn. *Phallus indusiatus*). Biomedicines.

[8] Kumar Y., Xu B. (2025). New Insights into Chemical Profiles and Health-Promoting Effects of Edible Mushroom *Dictyophora indusiata* (Vent Ex. Pers.) Fischer: A Review. J. Fungi.

[9] Oyetayo V.O., Dong C.-H., Yao Y.-J. (2009). Antioxidant and Antimicrobial Properties of Aqueous Extract from *Dictyophora indusiata*. Open Mycol. J..

[10] Deng C., Fu H., Teng L., Hu Z., Xu X., Chen J., Ren T. (2013). Anti-Tumor Activity of the Regenerated Triple-Helical Polysaccharide from *Dictyophora indusiata*. Int. J. Biol. Macromol..

[11] Fu H., Deng C., Teng L., Yu L., Su T., Xu X., Chen J., Yang C. (2015). Immunomodulatory Activities on RAW 264.7 Macrophages of a Polysaccharide from Veiled Lady Mushroom, *Dictyophora indusiata* (Higher Basidiomycetes). Int. J. Med. Mushrooms.

[12] Wang W., Song X., Gao Z., Zhao H., Wang X., Liu M., Jia L. (2019). Anti-Hyperlipidemic, Antioxidant and Organic Protection Effects of Acidic-Extractable Polysaccharides from *Dictyophora indusiata*. Int. J. Biol. Macromol..

[13] Kawagishi H., Ishiyama D., Mori H., Sakamoto H., Ishiguro Y., Furukawa S., Li J. (1997). Dictyophorines A and B, Two Stimulators of NGF-Synthesis from the Mushroom *Dictyophora indusiata*. Phytochemistry.

[14] Lee I.K., Yun B.S., Han G., Cho D.H., Kim Y.H., Yoo I.D. (2002). Dictyoquinazols A, B, and C, New Neuroprotective Compounds from the Mushroom *Dictyophora indusiata*. J. Nat. Prod..

[15] Zhang Y., Xun H., Gao Q., Qi F., Sun J., Tang F. (2023). Chemical Constituents of the Mushroom *Dictyophora indusiata* and Their Anti-Inflammatory Activities. Molecules.

[16] Yongxia Z., Jian X., Suyuan H., Aixin N., Lihong Z. (2020). Isolation and Characterization of Ergosterol from *Monascus* Anka for Anti-Lipid Peroxidation Properties. J. Mycol. Med..

[17] Sun P., Li W., Guo J., Peng Q., Ye X., Hu S., Liu Y., Liu W., Chen H., Qiao J. (2023). Ergosterol Isolated from *Antrodia camphorata* Suppresses LPS-Induced Neuroinflammatory Responses in Microglia Cells and ICR Mice. Molecules.

[18] Sillapachaiyaporn C., Mongkolpobsin K., Chuchawankul S., Tencomnao T., Baek S.J. (2022). Neuroprotective Effects of Ergosterol against TNF-α-Induced HT-22 Hippocampal Cell Injury. Biomed. Pharmacother..

[19] Mahasuari N.P.S., Paramita N.L.P.V., Yadnya Putra A.A.G.R. (2020). Effect of Methanol Concentration as a Solvent on Total Phenolic and Flavonoid Content of Beluntas Leaf Extract (*Pulchea indica* L.). J. Pharm. Sci. Appl..

[20] Smiderle F.R., Morales D., Gil-Ramírez A., de Jesus L.I., Gilbert-López B., Iacomini M., Soler-Rivas C. (2017). Evaluation of Microwave-Assisted and Pressurized Liquid Extractions to Obtain β-d-Glucans from Mushrooms. Carbohydr. Polym..

[21] Sun Y., He H., Wang Q., Yang X., Jiang S., Wang D. (2022). A Review of Development and Utilization for Edible Fungal Polysaccharides: Extraction, Chemical Characteristics, and Bioactivities. Polymers.

[22] Venkatesan T., Choi Y.W., Kim Y.K. (2019). Impact of Different Extraction Solvents on Phenolic Content and Antioxidant Potential of *Pinus densiflora* Bark Extract. Biomed. Res. Int..

[23] Dupont S., Fleurat-Lessard P., Cruz R.G., Lafarge C., Grangeteau C., Yahou F., Gerbeau-Pissot P., Abrahão Júnior O., Gervais P., Simon-Plas F. (2021). Antioxidant Properties of Ergosterol and Its Role in Yeast Resistance to Oxidation. Antioxidants.

[24] Pan M., Chitra E., Ghosh S. (2019). Roles of Cholinergic System in Cognitive Dysfunction. Eur. J. Pharm. Med. Res..

[25] Naveen S., Siddalingaswamy M., Singsit D., Khanum F. (2013). Anti-Depressive Effect of Polyphenols and Omega-3 Fatty Acid from Pomegranate Peel and Flax Seed in Mice Exposed to Chronic Mild Stress. Psychiatry Clin. Neurosci..

[26] Maneenet J., Daodee S., Monthakantirat O., Boonyarat C., Khamphukdee C., Kwankhao P., Pitiporn S., Awale S., Chulikhit Y., Kijjoa A. (2019). Kleeb Bua Daeng, a Thai Traditional Herbal Formula, Ameliorated Unpredictable Chronic Mild Stress-Induced Cognitive Impairment in ICR Mice. Molecules.

[27] Kraeuter A.-K., Guest P.C., Sarnyai Z. (2019). The Y-Maze for Assessment of Spatial Working and Reference Memory in Mice. Methods Mol. Biol..

[28] Gumuslu E., Mutlu O., Sunnetci D., Ulak G., Celikyurt I.K., Cine N., Akar F., Savlı H., Erden F. (2014). The Antidepressant Agomelatine Improves Memory Deterioration and Upregulates CREB and BDNF Gene Expression Levels in Unpredictable Chronic Mild Stress (UCMS)-Exposed Mice. Drug Target. Insights.

[29] Alawiyah K., Juliandi B., Boediono A., Sasai N. (2020). Oral Administration of Incense Resin (*Styrax benzoin*) Extract Enhances Spatial Learning, Memory, and Dendrite Complexity of Mice. Braz. Arch. Biol. Technol..

[30] Chhillar R., Dhingra D. (2013). Antidepressant-like Activity of Gallic Acid in Mice Subjected to Unpredictable Chronic Mild Stress. Fundam. Clin. Pharmacol..

[31] Zhang J., Hu T., Wang Y., Zhang X., Zhang H., Lin J., Tang X., Liu X., Chen M., Khan N.U. (2022). Investigating the Neurotoxic Impacts of Arsenic and the Neuroprotective Effects of *Dictyophora polysaccharide* Using SWATH-MS-Based Proteomics. Molecules.

[32] Zhao R., Master B.Q., Master B.M., Cai Y. (2019). Improving Activity of *Lycium barbarum* Polysaccharide on Depressive Mice Induced by Reserpine. Iran. J. Pharm. Res..

[33] Nazir Y., Linsaenkart P., Khantham C., Chaitep T., Jantrawut P., Chittasupho C., Rachtanapun P., Jantanasakulwong K., Phimolsiripol Y., Sommano S.R. (2021). High Efficiency In Vitro Wound Healing of *Dictyophora indusiata* Extracts via Anti-Inflammatory and Collagen Stimulating (MMP-2 Inhibition) Mechanisms. J. Fungi.

[34] Kiliç I., Yeşiloğlu Y. (2013). Spectroscopic Studies on the Antioxidant Activity of P-Coumaric Acid. Spectrochim. Acta A Mol. Biomol. Spectrosc..

[35] Zhang J., Shi R., Li H., Xiang Y., Xiao L., Hu M., Ma F., Wah C., Huang Z. (2016). Antioxidant and Neuroprotective Effects of *Dictyophora indusiata* Polysaccharide in *Caenorhabditis elegans*. J. Ethnopharmacol..

[36] Seo H.Y., Lee S.H., Lee J.H., Hwang J.S., Kim M.K., Jang B.K. (2020). Kahweol Activates the Nrf2/HO-1 Pathway by Decreasing Keap1 Expression Independently of P62 and Autophagy Pathways. PLoS ONE.

[37] Liu S., Pi J., Zhang Q. (2022). Signal Amplification in the KEAP1-NRF2-ARE Antioxidant Response Pathway. Redox Biol..

[38] Gan L., Johnson J.A. (2014). Oxidative Damage and the Nrf2-ARE Pathway in Neurodegenerative Diseases. Biochim. Biophys. Acta.

[39] Yu C., Xiao J.-H. (2021). The Keap1-Nrf2 System: A Mediator between Oxidative Stress and Aging. Oxidative Med. Cell. Longev..

[40] Tu W., Wang H., Li S., Liu Q., Sha H. (2019). The Anti-Inflammatory and Anti-Oxidant Mechanisms of the Keap1/Nrf2/ARE Signaling Pathway in Chronic Diseases. Aging Dis..

[41] Ruksiriwanich W., Khantham C., Linsaenkart P., Chaitep T., Rachtanapun P., Jantanasakulwong K., Phimolsiripol Y., Režek Jambrak A., Nazir Y., Yooin W. (2022). Anti-inflammation of Bioactive Compounds from Ethanolic Extracts of Edible Bamboo Mushroom (*Dictyophora indusiata*) as Functional Health Promoting Food Ingredients. Int. J. Food Sci. Tech..

[42] Lian J., Li K., Gao J., Tan X., Yang Z. (2019). Legumain Acts on Neuroinflammatory to Affect CUS-Induced Cognitive Impairment. Behav. Brain Res..

[43] Zhao J., Bi W., Xiao S., Lan X., Cheng X., Zhang J., Lu D., Wei W., Wang Y., Li H. (2019). Neuroinflammation Induced by Lipopolysaccharide Causes Cognitive Impairment in Mice. Sci. Rep..

[44] Bollinger J.L., Bergeon Burns C.M., Wellman C.L. (2016). Differential Effects of Stress on Microglial Cell Activation in Male and Female Medial Prefrontal Cortex. Brain Behav. Immun..

[45] Liu Y., Zhang H., Li Y., Zha H., Gao Y., Chen H., Wang Y., Zhou T., Deng C. (2024). *Dictyophora indusiata* Polysaccharide Mediates Priming of the NLRP3 Inflammasome Activation via TLR4/ NF-κB Signaling Pathway to Exert Immunostimulatory Effects. J. Appl. Biomed..

[46] Wang Y., Lai L., Teng L., Li Y., Cheng J., Chen J., Deng C. (2019). Mechanism of the Anti-Inflammatory Activity by a Polysaccharide from *Dictyophora indusiata* in Lipopolysaccharide-Stimulated Macrophages. Int. J. Biol. Macromol..

[47] Singleton V.L., Rossi J.A. (1965). Colorimetry of Total Phenolics with Phosphomolybdic-Phosphotungstic Acid Reagents. Am. J. Enol. Vitic..

[48] Laczkó-Zöld E., Komlósi A., Ülkei T., Fogarasi E., Croitoru M., Fülöp I., Domokos E., Ştefănescu R., Varga E. (2018). Extractability of Polyphenols from Black Currant, Red Currant and Gooseberry and Their Antioxidant Activity. Biol. Futur..

[49] Ibrahim H. (2014). Effect of β-Glucan Extracted from *Saccharomyces cerevisiae* on Angiogenesis. Master’s Thesis.

[50] Mamah B. (2017). Development of Cosmetic Product from Leaves of *Moringa oleifera* Lam. Collected in Sripoom Community in Thonburi Area. Isan J. Pharm. Sci..

[51] Tailor C.S., Goyal A. (2014). Antioxidant Activity by DPPH Radical Scavenging Method of *Ageratum conyzoides* Linn. Leaves. Am. J. Ethnomed..

[52] Ellman G.L., Courtney K.D., Andres V., Featherstone R.M. (1961). A New and Rapid Colorimetric Determination of Acetylcholinesterase Activity. Biochem. Pharmacol..

[53] Ingkaninan K., Temkitthawon P., Chuenchom K., Yuyaem T., Thongnoi W. (2003). Screening for Acetylcholinesterase Inhibitory Activity in Plants Used in Thai Traditional Rejuvenating and Neurotonic Remedies. J. Ethnopharmacol..

[54] Mizuki D., Qi Z., Tanaka K., Fujiwara H., Ishikawa T., Higuchi Y., Matsumoto K. (2014). *Butea Superba*–Induced Amelioration of Cognitive and Emotional Deficits in Olfactory Bulbectomized Mice and Putative Mechanisms Underlying Its Actions. J. Pharmacol. Sci..

[55] Khamphukdee C., Turkmani I., Chotritthirong Y., Chulikhit Y., Boonyarat C., Sekeroglu N., Silva A.M.S., Monthakantirat O., Kijjoa A. (2022). Effects of the Bark Resin Extract of *Garcinia nigrolineata* on Chronic Stress-Induced Memory Deficit in Mice Model and the In Vitro Monoamine Oxidases and β-Amyloid Aggregation Inhibitory Activities of Its Prenylated Xanthone Constituents. Molecules.

