# Supplementary material for: Effects of Dictyophora indusiata on Cognitive Impairment and Associated Neurobiological Changes in Chronic Mild Stress Mouse Model
Source: Int J Mol Sci. 2026 Sep 9;27(18):8027. doi: 10.3390/ijms27188027 (PMC13606763; doi:10.3390/ijms27188027)
Supplement: Supplementary file 1 [file ijms-27-08027-s001.zip › ijms-4507436-supplementary.pdf]

Supplementary Materials

Effects of *Dictyophora indusiata* on Cognitive Impairment and Associated Neurobiological Changes in Chronic Mild stress Mouse Model

Jatuporn Prathumtet [1], Tantima Kumlung [2,3], Warisada Sila-On [2,4], Sureewan Duangjit [1,2], Rawiwun Kaewamatawong [1], Wiwat Pichayakorn [5], Yaowared Sumanont [6], Orawan Monthakantirat [6] and Utsana Puapermpoonsiri [1,2*]

[1] Faculty of Pharmaceutical Sciences, Ubon Ratchathani University, Ubon Ratchathani 34190, Thailand; jatuporn.pr.63@ubu.ac.th (J.P.); sureewan.d@ubu.ac.th (S.D.); rawiwun.k@ubu.ac.th (R.K.)

[2] Innovation in Drug and Extract of Agriculture (IDEA) Research Group, Ubon Ratchathani University, Ubon Ratchathani 34190, Thailand; tantima@tistr.or.th (T.K.); warisada.s@rsu.ac.th (W.S.-O.)

[3] Thailand Institute of Scientific and Technological Research (TISTR), Pathum Thani 12120, Thailand

[4] College of Pharmacy, Rangsit University, Pathum Thani 12000, Thailand

[5] Faculty of Pharmaceutical Sciences, Prince of Songkla University, Songkhla 90110, Thailand; wiwat.p@psu.ac.th.

[6] Faculty of Pharmaceutical Sciences, Khon Kaen University, Khon Kaen 40002, Thailand; yaosum@kku.ac.th (Y.S.); oramon@kku.ac.th (O.M.)

* Correspondence: utsana.p@ubu.ac.th; Tel.: +66-96-395-1987

1. Statistical Analysis Effect of D. indusiata on UCMS-induced cognitive impairment in mice

Table S1. One-way ANOVA of locomotor activity measured by total arm entries in the Y-maze test

Group comparison	ANOVA	
	P	*F* ($DF_{between\ group}$,$DF_{residual}$)
Locomotor activity		
non-stress group	Not significant	*F*(14,45)=0.950
UCMS + vehicle group		
UCMS + Vitamin E group		
UCMS + DI 50 group		
UCMS + DI 200 group		
UCMS + DI 800 group		

Table S2. Independent t-test and One-way analysis of variance (ANOVA) test of % alternation in the Y-maze test

Group comparison	ANOVA followed by Tukey's post hoc test	
	P	*F* ($DF_{between\ group}$,$DF_{residual}$)
Y-maze test		
non-stress group vs. UCMS + vehicle group	<0.001	*t*(18)=5.688
UCMS + vehicle group vs. UCMS + Vitamin E group	0.003	*F*(4,45)=6.074
UCMS + vehicle group vs. UCMS + DI 50 group	Not significant	
UCMS + vehicle group vs. UCMS + DI 200 group	Not significant	
UCMS + vehicle group vs. UCMS + DI 800 group	0.01	
UCMS + DI 50 group vs. UCMS + DI 200 group	Not significant	
UCMS + DI 50 group vs. UCMS + DI 800 group	0.02	

Table S3. Independent t-test and One-way analysis of variance (ANOVA) test of % discrimination index in the Novel Object Recognition Test (NORT)

Group comparison	ANOVA followed by Tukey's post hoc test	
	P	*F* ($DF_{between\ group}$,$DF_{residual}$)
NORT		
non-stress group vs. UCMS + vehicle group	<0.001	*t*(18)=8.502
UCMS + vehicle group vs. UCMS + Vitamin E group	<0.001	*F*(4,45)= 26.103
UCMS + vehicle group vs. UCMS + DI 50 group	0.082	
UCMS + vehicle group vs. UCMS + DI 200 group	0.001	
UCMS + vehicle group vs. UCMS + DI 800 group	<0.001	
UCMS + DI 50 group vs. UCMS + DI 200 group	Not significant	
UCMS + DI 50 group vs. UCMS + DI 800 group	<0.001	

Table S4. Two-way repeated-measures ANOVA of escape latency during the 5-day training phase of the Morris water maze (MWM) test

Group comparison	Two-way repeated-measures ANOVA followed by Tukey's post hoc test		
	P	Test of Within Subjects Effects	Test of Between Subjects Effects
		F (DF)	*F* (DF$_{between\ group}$,DF$_{residual}$)
Group*Day			
non-stress group vs. UCMS + vehicle group	<0.001	F(2.212)= 20	F(5,1)= 44.459
UCMS + vehicle group vs. UCMS + Vitamin E group	<0.001		
UCMS + vehicle group vs. UCMS + DI 50 group	<0.001		
UCMS + vehicle group vs. UCMS + DI 200 group	<0.001		
UCMS + vehicle group vs. UCMS + DI 800 group	<0.001		
UCMS + DI 50 group vs. UCMS + DI 200 group	Not significant		
UCMS + DI 50 group vs. UCMS + DI 800 group	Not significant		

Table S5. Independent t-test and One-way analysis of variance (ANOVA) test of time in target quadrant (test phase) in the MWM test

Group comparison	ANOVA followed by Tukey's post hoc test	
	P	*F* (DF$_{between\ group}$,DF$_{residual}$)
MWM test		
non-stress group vs. UCMS + vehicle group	<0.001	t(18)=25.653
UCMS + vehicle group vs. UCMS + Vitamin E group	<0.001	F(4,45)=76.252
UCMS + vehicle group vs. UCMS + DI 50 group	<0.001	
UCMS + vehicle group vs. UCMS + DI 200 group	<0.001	
UCMS + vehicle group vs. UCMS + DI 800 group	<0.001	
UCMS + DI 50 group vs. UCMS + DI 200 group	0.001	
UCMS + DI 50 group vs. UCMS + DI 800 group	<0.001	

2. Statistical Analysis Effect of D. indusiata on UCMS-induced oxidative stress in the mouse brain

Table S6. Independent t-test and One-way analysis of variance (ANOVA) test of SOD activity (U/mg protein) in SOD test

Group comparison	ANOVA followed by Tukey's post hoc test	
	P	*F* ($DF_{between\ group}$,$DF_{residual}$)
Frontal cortex		
non-stress group vs. UCMS + vehicle group	<0.001	*t*(8)=28.548
UCMS + vehicle group vs. UCMS + Vitamin E group	<0.001	*F*(4,20)=330.614
UCMS + vehicle group vs. UCMS + DI 50 group	Not significant	
UCMS + vehicle group vs. UCMS + DI 200 group	<0.001	
UCMS + vehicle group vs. UCMS + DI 800 group	<0.001	
UCMS + DI 50 group vs. UCMS + DI 200 group	<0.001	
UCMS + DI 50 group vs. UCMS + DI 800 group	<0.001	
Hippocampus		
non-stress group vs. UCMS + vehicle group	<0.001	*t*(8)=13.139
UCMS + vehicle group vs. UCMS + Vitamin E group	<0.001	*F*(4,20)=31.485
UCMS + vehicle group vs. UCMS + DI 50 group	Not significant	
UCMS + vehicle group vs. UCMS + DI 200 group	0.003	
UCMS + vehicle group vs. UCMS + DI 800 group	<0.001	
UCMS + DI 50 group vs. UCMS + DI 200 group	0.009	
UCMS + DI 50 group vs. UCMS + DI 800 group	<0.001	

Table S7. Independent t-test and One-way analysis of variance (ANOVA) test of CAT activity (U/mg protein) in CAT test

Group comparison	ANOVA followed by Tukey's post hoc test	
	P	*F* ($DF_{between\ group}$,$DF_{residual}$)
Frontal cortex		
non-stress group vs. UCMS + vehicle group	<0.001	*t*(8)=6.051
UCMS + vehicle group vs. UCMS + Vitamin E group	<0.001	*F*(4,20)=27.387
UCMS + vehicle group vs. UCMS + DI 50 group	0.002	
UCMS + vehicle group vs. UCMS + DI 200 group	<0.001	
UCMS + vehicle group vs. UCMS + DI 800 group	<0.001	
UCMS + DI 50 group vs. UCMS + DI 200 group	Not significant	
UCMS + DI 50 group vs. UCMS + DI 800 group	0.009	
Hippocampus		
non-stress group vs. UCMS + vehicle group	<0.001	*t*(8)=7.951
UCMS + vehicle group vs. UCMS + Vitamin E group	<0.001	*F*(4,20)=14.642
UCMS + vehicle group vs. UCMS + DI 50 group	Not significant	
UCMS + vehicle group vs. UCMS + DI 200 group	0.005	
UCMS + vehicle group vs. UCMS + DI 800 group	<0.001	
UCMS + DI 50 group vs. UCMS + DI 200 group	Not significant	
UCMS + DI 50 group vs. UCMS + DI 800 group	0.002	

Table S8. Independent t-test and One-way analysis of variance (ANOVA) test of MDA levels (nmol/mg protein) in lipid peroxidation test

Group comparison	ANOVA followed by Tukey's post hoc test	
	P	*F* ($DF_{between\ group}$,$DF_{residual}$)
Frontal cortex		
non-stress group vs. UCMS + vehicle group	<0.001	*t*(8)=-11.600
UCMS + vehicle group vs. UCMS + Vitamin E group	<0.001	*F*(4,20)=55.834
UCMS + vehicle group vs. UCMS + DI 50 group	Not significant	
UCMS + vehicle group vs. UCMS + DI 200 group	<0.001	
UCMS + vehicle group vs. UCMS + DI 800 group	<0.001	
UCMS + DI 50 group vs. UCMS + DI 200 group	<0.001	
UCMS + DI 50 group vs. UCMS + DI 800 group	<0.001	
Hippocampus		
non-stress group vs. UCMS + vehicle group	<0.001	*t*(8)=-11.057
UCMS + vehicle group vs. UCMS + Vitamin E group	<0.001	*F*(4,20)=46.294
UCMS + vehicle group vs. UCMS + DI 50 group	0.006	
UCMS + vehicle group vs. UCMS + DI 200 group	<0.001	
UCMS + vehicle group vs. UCMS + DI 800 group	<0.001	
UCMS + DI 50 group vs. UCMS + DI 200 group	0.003	
UCMS + DI 50 group vs. UCMS + DI 800 group	<0.001	

3. Statistical Analysis Effect of D. indusiata on the expression of genes involved in oxidative stress and inflammation in the mouse brain

Table S9. Independent t-test and One-way analysis of variance (ANOVA) test of Nrf2 gene expression in frontal cortex and hippocampus

Group comparison	ANOVA followed by Tukey's post hoc test	
	P	*F* ($DF_{between\ group}$,$DF_{residual}$)
Frontal cortex		
non-stress group vs. UCMS + vehicle group	<0.001	*t*(8)=14.475
UCMS + vehicle group vs. UCMS + Vitamin E group	<0.001	*F*(4,20)=91.715
UCMS + vehicle group vs. UCMS + DI 50 group	Not significant	
UCMS + vehicle group vs. UCMS + DI 200 group	<0.001	
UCMS + vehicle group vs. UCMS + DI 800 group	<0.001	
UCMS + DI 50 group vs. UCMS + DI 200 group	<0.001	
UCMS + DI 50 group vs. UCMS + DI 800 group	<0.001	
Hippocampus		
non-stress group vs. UCMS + vehicle group	<0.001	*t*(8)=39.552
UCMS + vehicle group vs. UCMS + Vitamin E group	<0.001	*F*(4,20)=371.495
UCMS + vehicle group vs. UCMS + DI 50 group	Not significant	
UCMS + vehicle group vs. UCMS + DI 200 group	<0.001	
UCMS + vehicle group vs. UCMS + DI 800 group	<0.001	
UCMS + DI 50 group vs. UCMS + DI 200 group	<0.001	
UCMS + DI 50 group vs. UCMS + DI 800 group	<0.001	

Table S10. Independent t-test and One-way analysis of variance (ANOVA) test of Keap1 gene expression in frontal cortex and hippocampus

Group comparison	ANOVA followed by Tukey's post hoc test	
	P	F ($DF_{between\ group}$,$DF_{residual}$)
Frontal cortex		
non-stress group vs. UCMS + vehicle group	<0.001	$t(8)$=-26.695
UCMS + vehicle group vs. UCMS + Vitamin E group	<0.001	$F(4,20)$=1301.246
UCMS + vehicle group vs. UCMS + DI 50 group	<0.001	
UCMS + vehicle group vs. UCMS + DI 200 group	<0.001	
UCMS + vehicle group vs. UCMS + DI 800 group	<0.001	
UCMS + DI 50 group vs. UCMS + DI 200 group	<0.001	
UCMS + DI 50 group vs. UCMS + DI 800 group	<0.001	
Hippocampus		
non-stress group vs. UCMS + vehicle group	<0.001	$t(8)$=-34.290
UCMS + vehicle group vs. UCMS + Vitamin E group	<0.001	$F(4,20)$=283.153
UCMS + vehicle group vs. UCMS + DI 50 group	<0.001	
UCMS + vehicle group vs. UCMS + DI 200 group	<0.001	
UCMS + vehicle group vs. UCMS + DI 800 group	<0.001	
UCMS + DI 50 group vs. UCMS + DI 200 group	<0.001	
UCMS + DI 50 group vs. UCMS + DI 800 group	<0.001	

Table S11. Independent t-test and One-way analysis of variance (ANOVA) test of IL-1β gene expression in frontal cortex and hippocampus

Group comparison	ANOVA followed by Tukey's post hoc test	
	P	F ($DF_{between\ group}$,$DF_{residual}$)
Frontal cortex		
non-stress group vs. UCMS + vehicle group	<0.001	$t(8)$=-20.051
UCMS + vehicle group vs. UCMS + Vitamin E group	<0.001	$F(4,20)$=96.254
UCMS + vehicle group vs. UCMS + DI 50 group	<0.001	
UCMS + vehicle group vs. UCMS + DI 200 group	<0.001	
UCMS + vehicle group vs. UCMS + DI 800 group	<0.001	
UCMS + DI 50 group vs. UCMS + DI 200 group	<0.001	
UCMS + DI 50 group vs. UCMS + DI 800 group	<0.001	
Hippocampus		
non-stress group vs. UCMS + vehicle group	<0.001	$t(8)$=-64.032
UCMS + vehicle group vs. UCMS + Vitamin E group	<0.001	$F(4,20)$=206.318
UCMS + vehicle group vs. UCMS + DI 50 group	0.015	
UCMS + vehicle group vs. UCMS + DI 200 group	<0.001	
UCMS + vehicle group vs. UCMS + DI 800 group	<0.001	
UCMS + DI 50 group vs. UCMS + DI 200 group	<0.001	
UCMS + DI 50 group vs. UCMS + DI 800 group	<0.001	

Table S12. Independent t-test and One-way analysis of variance (ANOVA) test of IL-6 gene expression in frontal cortex and hippocampus

Group comparison	ANOVA followed by Tukey's post hoc test	
	P	F ($DF_{between\ group}$,$DF_{residual}$)
**Frontal cortex**		
non-stress group vs. UCMS + vehicle group	<0.001	$t(8)=-12.434$
UCMS + vehicle group vs. UCMS + Vitamin E group	<0.001	$F(4,20)=79.255$
UCMS + vehicle group vs. UCMS + DI 50 group	0.008	
UCMS + vehicle group vs. UCMS + DI 200 group	<0.001	
UCMS + vehicle group vs. UCMS + DI 800 group	<0.001	
UCMS + DI 50 group vs. UCMS + DI 200 group	<0.001	
UCMS + DI 50 group vs. UCMS + DI 800 group	<0.001	
**Hippocampus**		
non-stress group vs. UCMS + vehicle group	<0.001	$t(8)=-33.300$
UCMS + vehicle group vs. UCMS + Vitamin E group	<0.001	F(4,20)=447.167
UCMS + vehicle group vs. UCMS + DI 50 group	<0.001	
UCMS + vehicle group vs. UCMS + DI 200 group	<0.001	
UCMS + vehicle group vs. UCMS + DI 800 group	<0.001	
UCMS + DI 50 group vs. UCMS + DI 200 group	<0.001	
UCMS + DI 50 group vs. UCMS + DI 800 group	<0.001	

Table S13. Independent t-test and One-way analysis of variance (ANOVA) test of TNF-α gene expression in frontal cortex and hippocampus

Group comparison	ANOVA followed by Tukey's post hoc test	
	P	F ($DF_{between\ group}$,$DF_{residual}$)
**Frontal cortex**		
non-stress group vs. UCMS + vehicle group	<0.001	$t(8)=-13.214$
UCMS + vehicle group vs. UCMS + Vitamin E group	<0.001	F(4,20)=109.773
UCMS + vehicle group vs. UCMS + DI 50 group	0.040	
UCMS + vehicle group vs. UCMS + DI 200 group	<0.001	
UCMS + vehicle group vs. UCMS + DI 800 group	<0.001	
UCMS + DI 50 group vs. UCMS + DI 200 group	<0.001	
UCMS + DI 50 group vs. UCMS + DI 800 group	<0.001	
**Hippocampus**		
non-stress group vs. UCMS + vehicle group	<0.001	$t(8)=-16.625$
UCMS + vehicle group vs. UCMS + Vitamin E group	<0.001	F(4,20)=94.388
UCMS + vehicle group vs. UCMS + DI 50 group	Not significant	
UCMS + vehicle group vs. UCMS + DI 200 group	<0.001	
UCMS + vehicle group vs. UCMS + DI 800 group	<0.001	
UCMS + DI 50 group vs. UCMS + DI 200 group	<0.001	
UCMS + DI 50 group vs. UCMS + DI 800 group	<0.001	
